# Comparison of right ventricular involvement in AL and transthyretin-type cardiac amyloidosis by cardiovascular magnetic resonance

**DOI:** 10.1186/1532-429X-11-S1-P54

**Published:** 2009-01-28

**Authors:** Evan Appelbaum, Caitlin Harrigan, Leah Biller, Warren J Manning, Frederick L Ruberg, Kenneth Tripp, Jeff Packman, Donna Grogan

**Affiliations:** 1 PERFUSE CMR Core Lab, Beth Israel Deaconess Medical Center, Boston, MA USA; 2 Amyloid Treatment and Research Program and Section of Cardiology, Department of Medicine, Boston University School of Medicine, Boston, MA USA; 3 FoldRx Pharmaceuticals, Inc, Cambridge, MA USA

**Keywords:** Right Ventricular, Cardiovascular Magnetic Resonance, Amyloidosis, Heart Failure Symptom, Cardiac Amyloidosis

## Background

Increased right ventricular (RV) wall thickness and RV dysfunction are adverse prognostic signs in primary (AL) cardiac amyloidosis and suggest more extensive disease. RV involvement in other forms of cardiac amyloidosis remains poorly understood. We sought to determine differences in RV involvement for those with transthyretin (TTR) type and light-chain (AL) type cardiac amyloidosis using cardiovascular magnetic resonance (CMR).

## Methods

We assessed a multi-center cohort of subjects with proven cardiac amyloidosis due to AL (n = 21, 14 M, age 61 +/- 11.5 yr) and TTR (n = 14 12 M, age 73 +/- 4.8 yr) using conventional cine SSFP CMR. RV thickening was defined as a wall thickness = 6 mm. Volumetric assessments of RV free wall mass, volumes and ejection fraction (EF) were measured by an independent core lab blinded to patient data.

## Results

The prevalence of RVH was greater in the TTR group (71% vs. 48% AL group, p = 0.01). Similarly, RV free wall mass was increased in the TTR subjects (55.4 ± 20.6 g vs 37.3 ± 15.4 g; p < 0.01). In addition, RV end-diastolic volume was greater in TTR amyloidosis (190 ± 39 g vs 138 ± 48 g; p < 0.01) while RVEF was lower in the TTR subjects (37 ± 11% vs 53 ± 13%; p < 0.001). There were no differences in the degree of heart failure symptoms in the TTR and AL groups: NHYA Class I: 4 vs 5; Class II/III: 10 vs 15; Class IV: 0 vs 1, respectively (p = 0.69).

## Conclusion

These data suggest that, while similar in clinical heart failure symptoms, TTR amyloidosis appears to have more prevalent and extensive RV involvement than AL. The clinical impact of RV involvement in TTR amyloidosis remains to be determined.

